# Air-drying of cells enables visualization of antiparallel microtubule overlaps in the spindle midzone

**DOI:** 10.1016/j.mex.2018.04.011

**Published:** 2018-04-24

**Authors:** Aya Ifuji, Takahisa Kuga, Yuji Nakayama

**Affiliations:** Department of Biochemistry & Molecular Biology, Kyoto Pharmaceutical University, Kyoto 607-8414, Japan

**Keywords:** Air-drying method, Anaphase, Antiparallel microtubule overlap, Immunofluorescence staining, Microtubule, Midzone, Mitosis, Telophase

## Abstract

Immunofluorescence staining is used extensively to examine various types of cellular events. However, even when an antibody can detect its epitopes in western blotting, it sometimes fails to detect its epitopes when used for immunofluorescence staining. One example is the antiparallel microtubule overlaps in the anaphase and telophase spindle midzone, which functions as a signaling scaffold for cleavage furrow specification. It has been believed that it cannot be visualized by immunofluorescence staining due to the highly dense structure of microtubule overlaps (Ifuji et al., 2017). Here, we show a simple method for visualization of antiparallel microtubule overlaps in the anaphase and telophase spindle midzone.

• Air-drying cells before fixation enables visualization of antiparallel microtubule overlaps in the anaphase and telophase spindle midzone, which cannot be visualized by the conventional method.
• Simple method that requires minimal usage of equipment.
• Commonly used anti-tubulin antibodies can be used in this method.

## Graphical abstract

**Figure:**
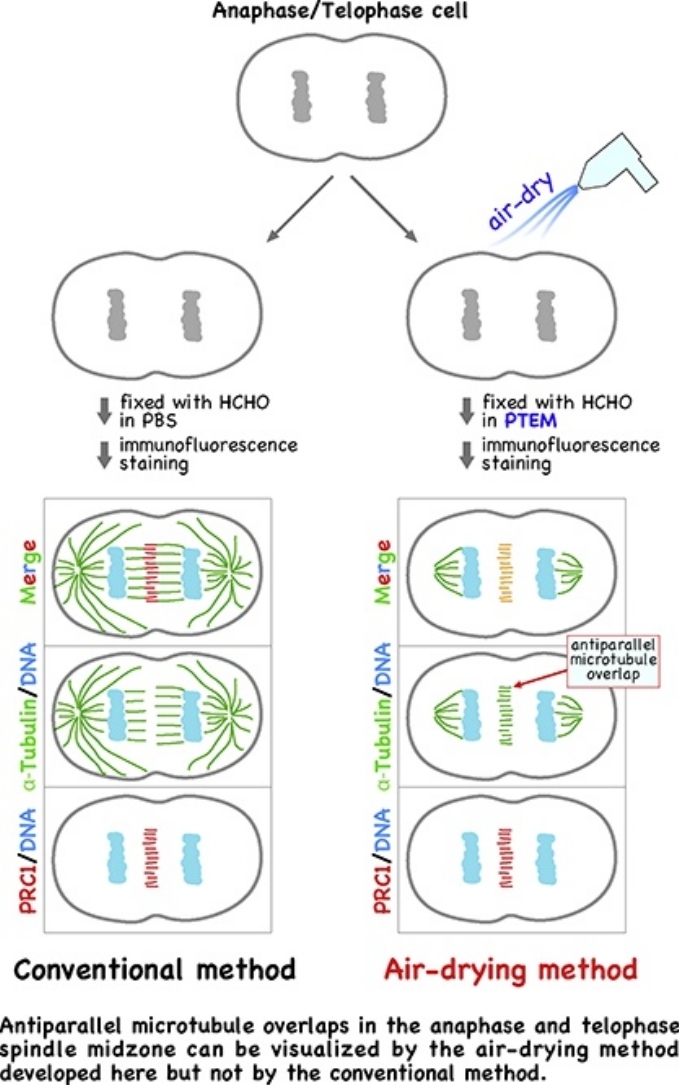

**Specifications Table**

**Table:** 

Subject area	• Biochemistry, Genetics, and Molecular Biology
More specific subject area	Cell biology, cell division
Method name	Air-drying method
Name and reference of original method	
Resource availability	

## Background

Immunofluorescence staining is used extensively to examine various types of cellular events related to the sub-cellular localization of proteins, shape of organelles, cell cycle stage, activation of proteins, protein-protein interactions, and so on. Antibodies with high affinity to their epitopes are useful for this purpose; however, this technique does not always achieve the intended goal. Even when an antibody can detect its epitopes in western blotting, it sometimes fails to detect its epitopes when used for immunofluorescence staining, possibly through structural issues.

Microtubules play important roles in various cellular situations, including in mitotic cells, where the polymerization and depolymerization dynamics of microtubules are essential for their function [2]. Their dynamics and stabilities depend on microtubule populations. It is difficult to examine their differences by western blotting using total α- or β-tubulin as a marker, since all populations of microtubules are combined into a single lysate. Tubulin post-translational modifications have been reported; they include phosphorylation, polyglutamylation, acetylation, tyrosination and detyrosination [3]. If posttranslational modifications that represent microtubule populations would be identified, it may be possible to have quantitative data by western blotting. If not, immunofluorescence staining of cells enables the examination of the differences in dynamics and stabilities of microtubules. For example, stable microtubules can be detected by immunofluorescence staining after cold treatment, which is known to disrupt unstable microtubules [4].

The anaphase and telophase spindle midzone includes antiparallel microtubule overlaps and functions as a signaling scaffold for cleavage furrow specification [5]. Thus, investigation of the formation and regulation of antiparallel microtubule overlaps in the anaphase and telophase midzone provides insights into the regulation of cytokinesis. Since posttranslational modifications that represent the antiparallel microtubule overlaps have never been identified, it may be impossible to have quantitative data by western blotting. In addition, it is hard to synchronize all cells in anaphase and telophase, which is required for preparation of the lysate. We previously developed the method to achieve anaphase and telophase-enriched populations by brief treatment of cells with the lower concentration of nocodazole and the myosin II inhibitor blebbistatin [6]. However, less than 50% of cells could be synchronized in anaphase and telophase. Thus, immunofluorescence staining is useful to analyze antiparallel microtubule overlaps in the anaphase and telophase midzone. Antiparallel microtubule overlaps have a highly dense structure; therefore, it has been believed that they cannot be visualized by immunofluorescence staining [1,7,8].

Here, we show a simple and fast method for visualization of antiparallel microtubule overlaps, which is not visualized by the conventional staining method, in the anaphase and telophase midzone with commonly used antibodies.

## Method details

**Fig. 1:**
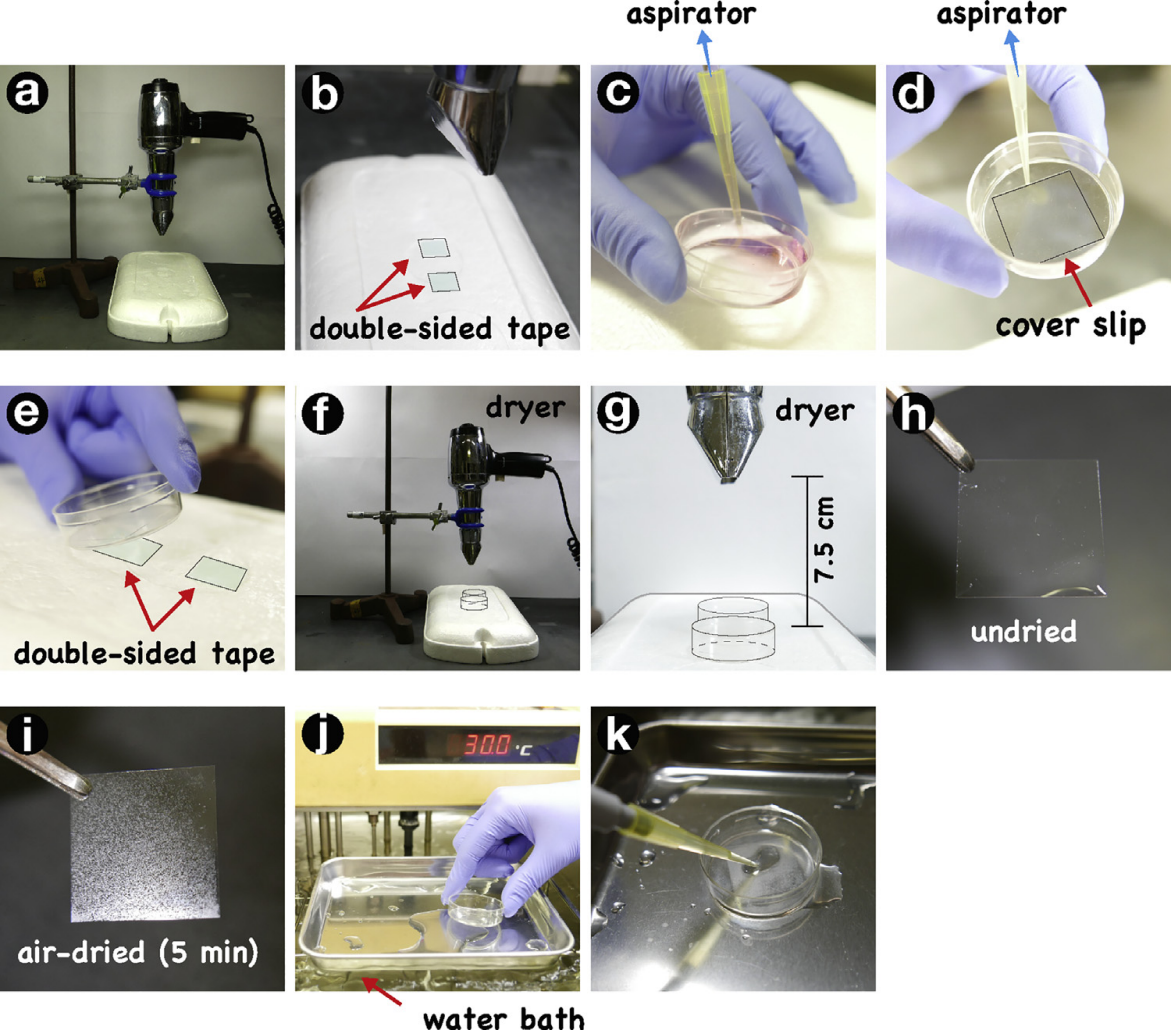
Schematic representation of the air-drying method.
2) Almost completely remove the culture medium from the cells on a cover slip by suction (Fig. 1c, d).
3) Stick the culture dish on the double-sided tape, which prevents the dish from being blown away by the hair dryer (Fig. 1e, f).
4) Air-dry the cells with cold air from the hair dryer for 5 min, after which the cells will appear to have dried (Fig. 1h, i). The time required to dry the cells depends on the strength of the air and the distance between the hair dryer and the cells. It is necessary to adjust this protocol to the particular equipment you are using.
5) Fix the cells with pre-warmed PTEMF buffer (2 mM PIPES [pH 6.8], 0.2% Triton X-100, 10 mM EGTA, 1 mM MgCl_2_, 4% formaldehyde) for 20 min on a water bath warmed at 30 °C. We usually place a metal tray on a water bath set at 30 °C and put the culture dishes on the tray. It is important to add a small amount of water to this metal tray in order to transfer the heat efficiently from the water bath to the culture dishes (Fig. 1j, k).
6) Wash the cells with PBS(−) and then stain the cells by the standard protocol with a commonly used anti-tubulin antibody as follows: The fixed cells were permeabilized with PBS(−) containing 0.1% saponin and 3% bovine serum albumin, and then cells were incubated with first and second antibodies for 1 h each [9].

To visualize antiparallel microtubule overlaps in the anaphase and telophase midzone, air-dry cultured cells and then fix these cells with PTEMF buffer (2 mM PIPES [pH 6.8], 0.2% Triton X-100, 10 mM EGTA, 1 mM MgCl_2_, 4% formaldehyde) [9]. The fixed cells can be subjected to standard immunofluorescence staining with commonly used anti-tubulin antibodies. The protocol is as follows:

1) Prepare the equipment as shown in Fig. 1a. Stick two pieces of double-sided tape for two dishes to a board made of expanded polystyrene, which is used to prevent cells from cooling (Fig. 1b). Place a hair dryer fixed in a stand at approximately 7.5 cm above the expanded polystyrene board (Fig. 1g). The height of a hair dryer should be adjusted on the basis of strength of air flow.

## Additional information

To optimize this method, we compared different fixing solutions (Table 1). The cells were air-dried after the removal of the culture medium and then fixed with PTEM buffer containing 0.5, 1 and 4% formaldehyde for 20 min at 30 °C. Although 0.5% formaldehyde did not fix the cells and many mitotic cells detached from the culture dish, 1, and 4% formaldehyde gave a similar staining pattern, indicating that a concentration of formaldehyde in the range of 1–4% does not affect the staining of antiparallel microtubule overlaps.

**Table 1:** Effects of fixing solution. HeLa S3 cells were air-dried and then fixed with the indicated fixing solutions.

Immunofluorescence staining of proteins in the nuclear envelope and centrosome sometimes requires the fixation of cells with methanol. We, thus, examined whether methanol fixation could be used for visualization of antiparallel microtubule overlaps. However, no microtubule filament was observed in the anaphase and telophase cells, indicating that it is impractical for this purpose. While methanol fixation sometimes fails to fix some proteins, this can be improved by using 4% formaldehyde in the presence of 20% methanol, which enables visualization of some nuclear and centrosomal proteins [10]. However, this fixation protocol was also impractical for visualization of antiparallel microtubule overlaps, although staining of the Flemming body was improved.

The temperature affects the stability of microtubules. In addition, the temperature of the fixation step can affect the time required for complete fixation [11] and also immunofluorescence staining. The cells were air-dried after the removal of the culture medium and then fixed with PTEM buffer containing 4% formaldehyde on a water bath set at 15, 20, 25, 30, or 37 °C (Table 2). Unexpectedly, this difference in temperature at the fixation step had a large effect on the result. Fixation at 15, 20, 25, and 37 °C did not enable visualization of antiparallel microtubule overlaps, suggesting that temperature at the fixation step should be controlled at approximately 30 °C.

**Table 2:** Effects of temperature during fixation. HeLa S3 cells were air-dried and then fixed for 5 min with PTEMF buffer at the indicated temperatures.

Given that the dynamics of polymerization and depolymerization and the stabilities of microtubules depend on microtubule populations, cold treatment just before air-drying the cells should depolymerize unstable microtubules, and the remaining, stable microtubules can then be visualized. The cells (still in their culture medium) were placed on ice for 3, 10, or 20 min before air-drying and the subsequent fixation step with PBS(−) containing 4% formaldehyde was performed at 30 °C or on ice (Table 3). As a result, antiparallel microtubule overlaps could not be visualized, indicating that cold treatment before air-drying the cells does not improve the result and that it is important to prevent the cells from being exposed to low temperatures before air-drying.

**Table 3:** Effects of cold treatment before air-drying the cells. HeLa S3 cells were placed on ice for the indicated times, air-dried, and fixed with PBS(−) containing 4% formaldehyde.

Lastly, we examined whether fetal bovine serum (FBS) concentration during maintenance of the cells affected visualization of antiparallel microtubule overlaps. However, we observed no difference in their visualization irrespective of FBS concentration, suggesting that the culture condition does not affect their visualization.

## Method validation

**Fig. 2:**
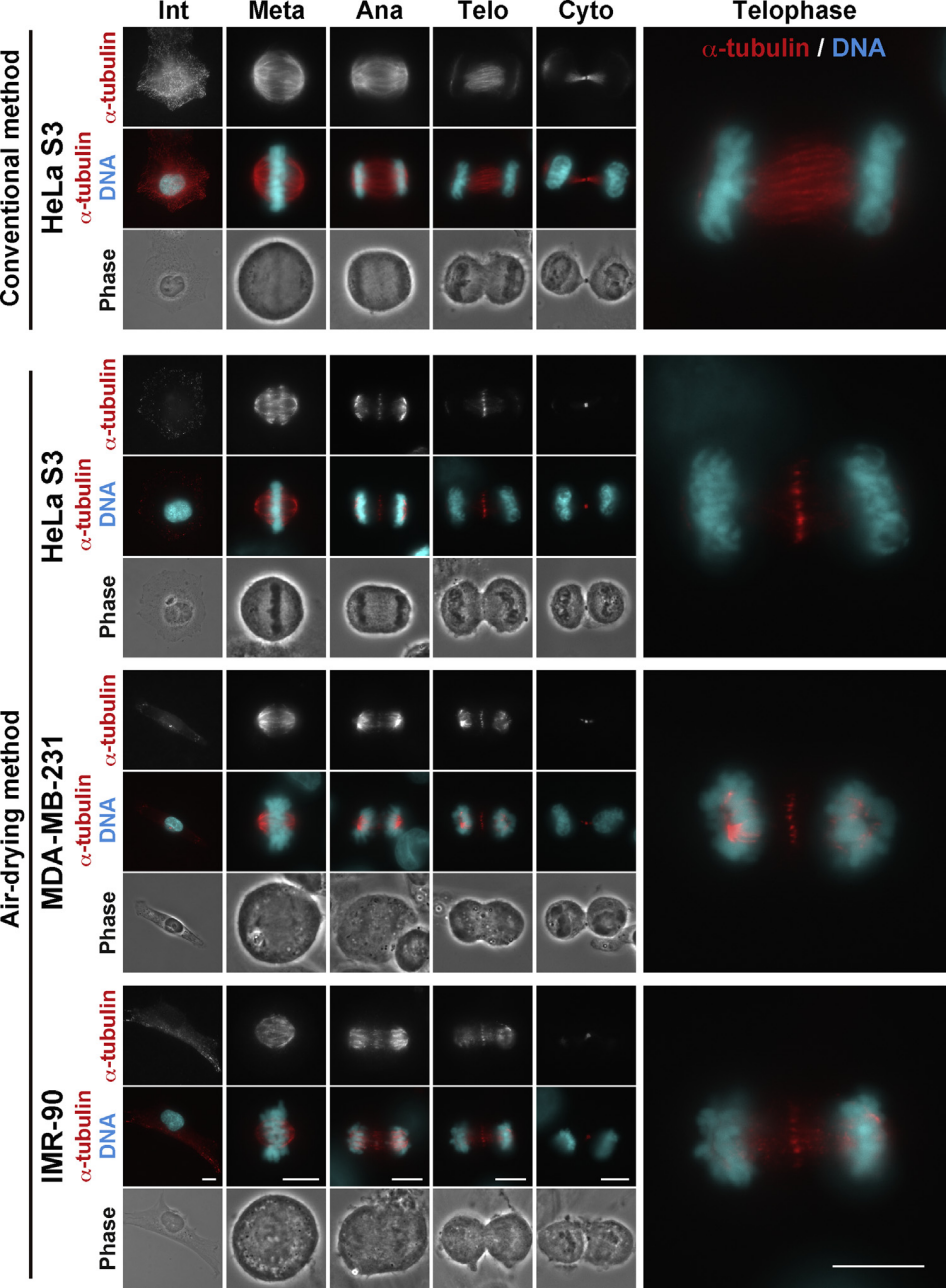
Visualization of antiparallel microtubule overlaps in the anaphase and telophase spindle midzone. HeLa S3, MDA-MB-231, and IMR-90 cells were air-dried, fixed with PTEMF buffer, and stained for α-tubulin (red) and DNA (cyan). As a comparison, the conventional method, that is, HeLa S3 cells fixed with PTEMF buffer without air-drying, was used. Merged images of telophase cells were magnified. Scale bars, 10 μm.

To validate the air-drying method optimized in this study, 3 cell lines were subjected to the air-drying method. As expected, antiparallel microtubule overlaps in the anaphase and telophase midzone were visualized in HeLa S3, MDA-MB-231, and IMR-90 cells (Fig. 2), suggesting that this method can be applicable to a variety of cell lines. By using this method, we have already reported why air-drying can visualize antiparallel microtubule overlaps and the effect of some kinase inhibitors on the formation of antiparallel microtubule overlaps [1].
